# Survival of people living with HIV who defaulted from tuberculosis treatment in a cohort, Recife, Brazil

**DOI:** 10.1186/s12879-016-2127-5

**Published:** 2017-02-10

**Authors:** R Cunha, M Maruza, UR Montarroyos, I Coimbra, D de B Miranda-Filho, M de F Albuquerque, HR Lacerda, RAA Ximenes

**Affiliations:** 1 Department of Clinical Medicine, Universidade de Pernambuco, Rua Arnóbio Marques, 310 –Santo Amaro, Recife, PE CEP: 50100-130 Pernambuco Brazil; 2 Hospital Correia Picanço – HCP - Health State Department, Rua Padre Roma, 149, Tamarineira, Recife, PE CEP: 52050-150 Pernambuco Brazil; 3 Hospital Universitário Oswaldo Cruz- HUOC- UPE, Rua Arnóbio Marques, 310 –Santo Amaro, Recife, PE CEP: 50100-130 Pernambuco Brazil; 4 Aggeu Magalhães Research Center/Fiocruz, Av Moraes Rego, s/n – Campos da UFPE - Cidade Universitária, Recife, PE CEP: 50670420 Pernambuco Brazil; 5 Post-Graduation Program in Tropical Medicine – Universidade Federal de Pernambuco, Hospital das Clínicas - Bl. A - Térreo do HC/UFPE, Av. Prof. Moraes Rego - s/n. - Cidade Universitária, Recife, PE CEP: 50670-901 Pernambuco Brazil

**Keywords:** Survival, Tuberculosis, Treatment, Default, Death

## Abstract

**Background:**

Tuberculosis is a serious public health problem worldwide. It is the leading cause of death amongst people living with HIV, and default from tuberculosis (TB) treatment in people living with HIV increases the probability of death. The aim of this study was to estimate the survival probability of people living with HIV who default treatment for TB compared to those who complete the treatment.

**Methods:**

This was a longitudinal cohort study of people living with HIV, from June 2007 to December 2013 with two components: a retrospective (for those who started tuberculosis treatment before 2013 for whom failure (death) or censoring occurred before 2013), and prospective (those who started tuberculosis treatment at any time between 2007 and June 2013 and for whom death or censoring occurred after the beginning of 2013), at two referral hospitals for people living with HIV (Correia Picanço Hospital - HCP and at Hospital Universitário Oswaldo Cruz – HUOC), in Recife/PE. A total of 317 patients who initiated TB treatment were studied. Default from TB treatment was defined as any patient who failed to attend their pre-booked return appointment at the health center for more than 30 consecutive days, in accordance with Brazilian Ministry of Health recommendations.

**Results:**

From a cohort of 2372 people living with HIV we analyzed 317 patients who had initiated TB treatment. The incidence of death was 5.6 deaths per 100 persons per year (CI 95% 4.5 to 7.08). Independent factors associated with death: default from TB treatment 3.65 HR (95% CI 2.28 to 5.83); CD4 < 200 cells/mm^3^ 2.39 HR (95% CI 1.44 to 3.96); extrapulmonary tuberculosis 1.56 HR (95% CI 0.93 to 2.63); smoking 2.28 HR (95% CI 1.33 to 3.89); alcohol light 0.13 HR (95% CI 0.03 to 0.56).

**Conclusion:**

The probability of death in people living with HIV who default TB treatment is approximately four times greater when compared to those who do not default from treatment.

## Background

Tuberculosis (TB) is a serious public health problem worldwide [1–3], and is the leading cause of morbidity and mortality in people living with HIV [4–7], who are 21 to 34 times more likely to develop TB when compared to those without HIV [2]. The pulmonary and extrapulmonary forms of tuberculosis are the most common opportunistic infections in people living with HIV [8, 9]. The interaction between Mycobacterium tuberculosis and HIV can cause elevation of viral load and decrease of CD4 + T lymphocytes count, leading to accelerated decline in the immune function and decreased survival [10] People living with HIV have been considered as being more likely to have unfavorable treatment outcomes of TB treatment, due to the complexity of the management of co-infection and to behavioral factors of this population [11]. Although almost all patients who receive TB treatment are cured, those living with HIV and undergoing concomitant treatment for HIV infection and TB present a number of challenges, amongst which are: the long duration of therapy, the dosing frequency, the potential complex drug interaction and toxicity of the two therapies [1, 12–14]. Therefore, numerous factors may influence the risk of defaulting TB treatment and death in HIV-infected individuals. Some authors found an association between defaulting from TB treatment and male sex, smoking, lymphocyte CD4 count <200 cells per mm3, low salaries, alcohol use, previous default from TB treatment, age of 35–49 years old, illiteracy and previous treatment for TB [15–18] Tuberculosis is the leading cause of death in people living with HIV, causing a great impact on the survival of this population. In turn, default from treatment features amongst the leading causes of TB treatment failure in people living with HIV, and may therefore be associated with a higher mortality rate within this group [7, 15, 19]. This aim of this study was to compare the survival probability of people living with HIV who defaulted treatment for TB with those who did not default treatment.

## Methods

This study is nested in the AIDS Cohort of the state of Pernambuco (Cohort AIDS-PE), a clinical cohort that has collected information of more than 2300 patients with HIV/AIDS attending two reference centers for the care of HIV/AIDS patients in Recife, Brazil. The study design was a longitudinal cohort with two components: retrospective, (June 2007 to December 2012) and prospective (January 2013 to December 2013). As we started this investigation in the beginning of 2013, people living with HIV who started tuberculosis treatment before 2013 and for whom failure (death) or censoring occurred before 2013, the study was retrospective. For those who started tuberculosis treatment at any time between June 2007 and June 2013 and for whom death or censoring occurred after the beginning of 2013, the study was prospective. The same selection procedures were used in the retrospective and prospective arm of the study. Patients attending two reference centers for the treatment of HIV (Hospital Correia Picanço and Hospital Universitário Oswaldo Cruz) were enrolled in the study at the time they were notified to the Surveillance System for Infectious Diseases (SINAN/MS) as having tuberculosis. Registering the case in the SINAN/MS is a prerequisite for initiation of TB treatment. They were then invited to participate in the study and after reading and signing the Informed Consent (IC) forms, they were interviewed by previously trained staff using a standardized questionnaire. Recruitment loss ocurred when the patient was notified to the Surveillance System for Infectious Diseases (SINAN/MS) as having tuberculosis but was not included in the study. Recruitment loss occurred in the retrospective arm (*n* = 182) as well as in the prospective (*n* = 10).

The study population consisted of people living with HIV aged 18 and over who had initiated treatment for TB. In our study TB cases were considered as those diagnosed of TB by the attending physician (according to the Ministry of Health of Brazil guidelines, that is based on clinical findings, direct investigation of Acid-fast bacillus –AFB -smear and culture for M.tb).and notified to the (SINAN/MS). Default from TB treatment was defined as any patient who failed to attend their pre-booked return appointment at the health center for more than 30 consecutive days. The study excluded individuals who presented any change in the diagnosis of TB during follow-up, those with multidrug-resistant TB (MDR-TB) and pregnant women. The response variable of the study was the occurrence of all-cause mortality. Information on the occurrence of death was obtained from medical records and from the Mortality Information System, ascertained through record linkage. Death registration is compulsory in Brazil. There is no burial without a death certificate and the death certificate is the source of information to the mortality system. The Mortality Information System is a national and sub-national electronic system with all routine mortality data coded to International Coding Disease. Registration of deaths is managed by the Ministry of Health in Brazil.

The main exposure was the default from treatment for TB. Patients were classified as defaulters or non defaulters. Defaulters were further classified according to the moment in which default ocurred (first quarter: meaning the first three months after the start of tuberculosis treatment and the second quarter meaning four to six months after the start of tuberculosis treatment).

Co-variables were grouped into six categories: biological variables (gender and age); variables related to habits and lifestyle (smoking and drinking); socioeconomic factors (income, place of residence and marital status); clinical variables (body mass index [BMI] and anemia); HIV-related variables (presence of opportunistic infection, AIDS case, CD4 count and antiretroviral therapy [ART]); variables related to tuberculosis (place of treatment, form of TB and previous treatment). Amongst the lifestyle variables, smoking was considered as the habit of cigarette smoking and categorized as: non-smokers (those who had never smoked during their lifetime); former smokers (those who had given up smoking at least six months prior to enrolling on the study); smokers (those who were smoking at the time of enrollment or had stopped smoking during the previous six months). Alcohol consumption was regarded as consuming alcoholic beverages and categorized as: abstainer (has never drunk or drinks less than 8 units/year); light drinker (drinks twice a week, at maximum, not exceeding 10 units/month); moderate drinker (drinks at least 3 or 4 times per week, exceeding 5 units/day); heavy drinker (undergoing treatment for alcoholism). The CD4+ count was considered as that observed at the time of initiating TB treatment and categorized as: greater than or equal to 200 cells/mm3 and less than 200 cells/mm3. The clinical form of TB was defined as the organ or tissue affected by the disease and categorized as: pulmonary TB (disease restricted to the lungs); extrapulmonary TB (when the disease involved organs other than the lungs); disseminated TB (involvement of two non-contiguous sites).

### Statistical analysis

For the data analysis, we considered the alpha level = 5% and a confidence interval (CI) of 95%. In the survival analysis, the Kaplan-Meier method was used to compare the probability of survival between those who defaulted from tuberculosis treatment and those who did not and the median survival time, and the log-rank test to test the statistical significance of the difference between the curves and compare survival curves. The death rate was expressed in person/years; time was measured since the start of tuberculosis treatment until death or failure. The semi-parametric regression Cox model was adopted to identify factors associated with survival time, in which the hazard ratio [HR] was used as the measurement of association. The association between default from tuberculosis treatment and death was adjusted for confounding factors. Deaths were identified by pairing individuals from the cohort during the follow-up period and the Mortality Information System of Pernambuco (SIM-PE), using the probabilistic linkage program RecLink III [20]. For the statistical analysis in the survival model we used STATA 12.0. The data entry and double entry validation were performed in parallel with data collection, and the database was managed by the SQL Server 2000 (Microsoft), using GeneXus software (version 7.5).

### Ethical considerations

The study was approved by the Research Ethics Committee at HUOC/PROCAPE/UPE on 02/07/2014, Report n°: 706. 132/CAAE: 30621114.8.0000.5192 and by the Ethics Committee of the Universidade Federal de Pernambuco, registered in the National System on Research Ethics (SISNEP) FR-067 159/CAAE-0004.1.172.106-05/REGISTRATION CEP/Health Sciences Center/Universidade Federal de Pernambuco (UFPE) Report n° 254/05 and funded by the Ministry of Health and Ministry of Science and Technology - CNPq/MS-SCTIE-DECIT (Case 10567/2006-0).

## Results

**Fig. 1:**
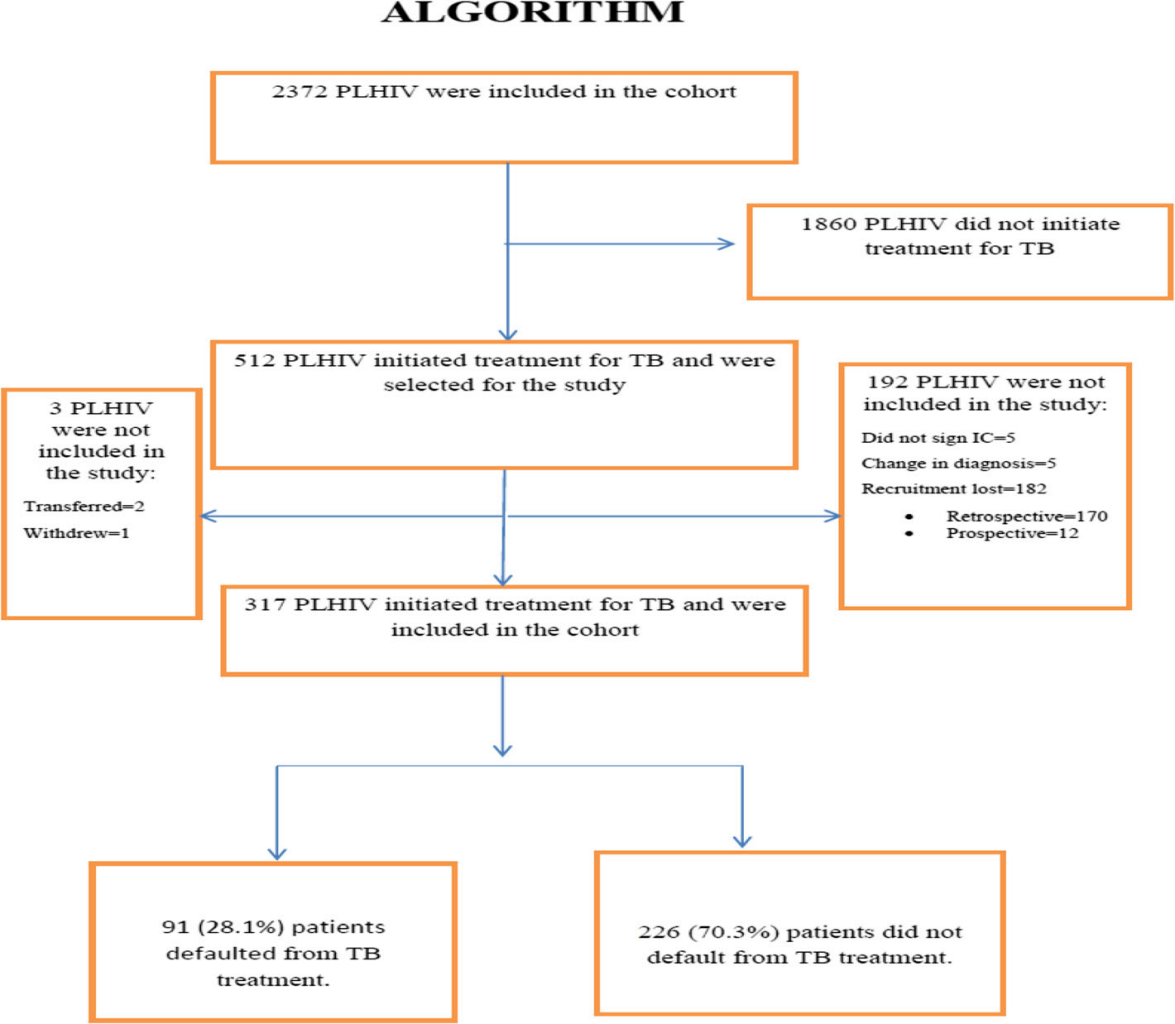
Algorithm demonstrating selection of study population

**Fig. 2:**
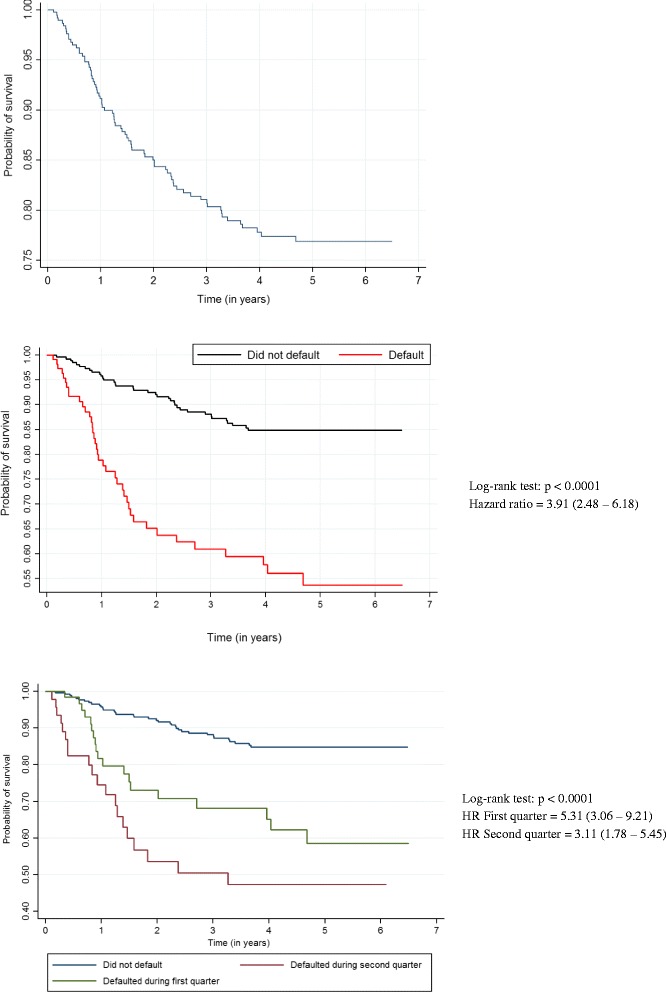
General survival curves and according to the condition of default in a cohort of people living with HIV who initiated the treatment for TB in Recife, Brazil

Out of 512 people living with HIV who initiated tuberculosis treatment, 317 were included in the study. Losses occurred because of: recruitment loss (*n* = 182, retrospective arm = 170 and prospective arm = 12), change in diagnosis (*n* = 5), not signing informed consent forms (*n* = 5) and loss to followup (*n* = 3). Of the 317 patients studied, 91 (28.7%) defaulted treatment for TB, while 226 (70.9%) did not (Fig. 1). A total of 59 individuals underwent further treatment for TB during follow-up, and contributed more than once to the cohort, thus resulting in a total of 375 treatment episodes for the entire cohort. From amongst these, there were 111defaults from TB treatment (29.6%) (Table 1), 47 (42.7%) during the first three months after the start of TB treatment and 63 (57.3%) between the fourth and sixth month after the start of TB treatment; the date of default of one patient was not recorded. From the 317 patients studied, 75 (23.6%) deaths were recorded during the study period. (Table 1) In the first year of follow-up there were 14 deaths (18.6%), in the second there were 25 (33.3%) and in the third year 36 (48%). Amongst the cases of death, 36 (48%) occurred in the group that did not default TB treatment, 20 (26.7%) in the group that defaulted treatment in the first quarter and 19 (25.3%) in the group that defaulted treatment in the second quarter. In the group that did not default treatment, 38.9% of the deaths occurred within two years of initiating treatment for TB, while the percentages of death for those who defaulted treatment in the first and second quarter were, respectively, 70% and 57.9%. (Table 2) The death rate amongst patients who defaulted TB treatment was 14.4 deaths per 100 person/years (95% CI: 10.5 to 19.7) and among patients who did not default treatment 3.4 deaths per 100 person/years (95% CI: 2.46 to 4.72) (Fig. 2). The probability of death for patients who defaulted treatment was 3.91 times greater (95% CI: 2.48 to 6.17) when compared to those who did not default treatment. Moreover, the probability of death was higher in those who defaulted from treatment early (Fig. 2).

**Table 1:** Characteristics of the study population

Characteristics	Statistics
Number of patients	317
Number of patients who defaulted treatment	91
Number of patients more than one treatment^a^	59
Length of follow-up in years (median and range)	3.54 (41 days; 6.5 years)
Number of deaths during treatment	75
Sex	
Male	227 (71.6%)
Female	90 (28.4%)
Age group	
< 30 years	66 (20.8%)
30 to 49 years	216 (68.1%)
≥ 50 years	35 (11.1%)
Use of ART	257 (81.1%)
CD4+ <200 cells/mm^3^	118 (37.2%)
Number of treatments^b^	375
Number of treatment defaults	111

^a^Number de patients who had more than one episode of treatment

^b^Number of treatments

**Table 2:** Association between death and defaulting from TB treatment^*^ in a cohort of PLHIV

	Time of default			
Time of death	Did not default	**Defaulted in the 1^st^ quarter	***Defaulted in the 2^nd^ quarter	Total
Less than 1 year	4 (11.1%)	4 (20.0%)	6 (31.6%)	14 (18.7%)
From 1 to 2 years	10 (27.8%)	10 (50.0%)	5 (26.3%)	25 (33.3%)
2 years and over	22 (61.1%)	6 (30%)	8 (42.1%)	36 (48.0%)
Total	36 (48.0%)	20 (26.7%)	19 (25.3%)	75 (100%)

* Chi Square Test *p*-value = 0.096 of the association between death and defaulting from TB treatment

** first three months after the start of tuberculosis treatment

*** four to six months after the start of tuberculosis treatment

In the univariate analysis, the following variables were significantly associated with death: age (30–49 years) (Table 3), heavy/dependent alcohol consumption and smoking (Table 4), a CD4 count <200 cells/mm3 (Table 5). In addition to treatment default, a CD4 count <200 cells/mm3, smoking, extrapulmonary TB and alcohol consumption all remained in the final model. (Table 6) Information on sputum smear was not available for 16.3% of the TB treatments; for those with available information, the percentage of TB treatments which had started with a positive sputum smear was 14.0%. Mortality was higher among those with a negative sputum smear (HR = 2.1, 95%-CI = 0.9–4.7, *p* = 0.075) than in those with a positive result. The probability of death was greater for defaulters in the the group with positive sputum smear (HR = 2.38, 95%-CI = 0.53–10.79), though it did not reach statistical significance; in the group with negative (HR = 3.74, 95%-CI = 1.88–7.46) and among those who did not have the sputum smear performed (HR = 3.30, 95%-CI = 1.63–6.67). We compared the group of patients not enrolled because they were not contactable by the researchers (*n* = 182) with the group of patients included in the study (*n* = 317) in relation to sex, age and default rate. The two groups were similar in relation to age and sex but the default rate was higher among those enrolled in the study.

**Table 3:** Association between death and biological and socioeconomic factors in a cohort of PLHIV

Variables	Deaths (number)	Incidence Rate (100 person/years)	HR (CI 95%)	*p*-value	*p*-value^a^
Biological^b^					
Sex					
Female	23	6.14 (4.08–9.25)	1.0	-	-
Male	52	5.44 (4.15–7.15)	0.88 (0.54–1.44)	0.625	0.231
Age group					
<30 years	24	8.38 (5.62–12.5)	1.0		
30 to 49 years	42	4.61 (3.40–6.23)	0.55 (0.33–0.90)	0.053	0.019
> 50 years	9	6.91 (3.60–13.3)	0.77 (0.35–1.66)	0.506	0.506
BMI					
Normal	32	4.35 (3.07–6.15)	1.0		
Malnourished	12	4.49 (2.54–7.97)	1.09 (0.56–2.11)	0.797	
Overweight/obese	6	3.23 (1.45–7.20)	0.78 (0.32–1.88)	0.591	
No information	25	17.8 (12.0–26.3)	3.75 (2.21–6.33)	0.000^†^	0.725
Anemia					
No	17	5.21 (3.24–8.39)	1.0	-	-
Yes	47	5.31 (4.00–7.07)	1.03 (0.59–1.79)	0.916	0.271
Socioeconomic^b^					
Origins					
Recife	29	5.29 (3.67–7.61)	1.0		
Metropolitan Region	36	6.80 (4.90–9.42)	1.29 (0.79–2.10)	0.309	
Interior of state	9	4.39 (2.28–8.44)	0.90 (0.42–1.90)	0.781	0.202
Race					
White	16	5.79 (3.54–9.44)	1.0		
Non-white	58	5.77 (4.45–7.46)	0.99 (0.57–1.72)	0.968	0.100
In a stable relationship					
No	38	5.25 (3.82–7.22)	1.0		
Yes	36	6.40 (4.61–8.87)	1.20 (0.76–1.90)	0.427	0.427
Schooling					
Illiterate	14	6.78 (4.01–11.5)	1.0		
From 1 to 9 years	43	6.13 (4.54–8.26)	0.88 (0.48–1.61)	0.677	
10 years or more	17	4.56 (2.83–7.33)	0.68 (0.34–1.39)	0.294	0.338
Employed					
Yes	11	4.57 (2.53–8.25)	1.0		
No	63	6.03 (4.71–7.71)	1.32 (0.69–2.50)	0.400	0.308
Income					
> 1 Minimum salary	23	5.01 (3.33–7.54)	1.0		

^a^Risk proportionality test (Schoenfeld residual)

^b^Biological and socioeconomic conditions when initiating treatment for TB

**Table 4:** Association between death and factors related to habits in a cohort of PLHIV

Variables	Deaths (number)	Incidence rate (100 person/years)	HR (CI 95%)	*p*-value	*p*-value^a^
Habits^b^					
Alcohol consumption					
No	58	5.97 (4.61–7.72)	1.0		
Yes. Light	2	1.09 (0.27–4.28)	0.18 (0.44–0.75)	0.018^†^	
Yes. Heavy/dependent	13	12.8 (7.41–22.0)	1.97 (1.08–3.61)	0.026^†^	0.912
Smoker^b^					
No	48	4.74 (3.57–6.29)	1.0		
Yes	26	9.47 (6.45–13.9)	1.88 (1.17–3.03)	0.010^†^	0.304
Use of drugs^c^					
No	53	6.04 (4.61–7.91)	1.0		
Yes	21	5.13 (3.34–7.87)	0.83 (0.50–1.37)	0.458	0.586

^a^Proportionality test (Schoenfeld residual)

^b^Condition of habits when initiating treatment for TB

^c^Use of drugs (marihuana. cocaine. crack or glue) at some point in life

**Table 5:** Association between death and factors related to TB and HIV in a cohort of PLHIV

Variables	Deaths (number)	Incidence rate (100 person/years)	HR (CI 95%)	*p*-value	*p*-value^a^
Related to TB^b^					
Treatment site					
Out-patients	31	5.00 (3.51–7.11)	1.0	-	
Hospitalized	44	6.51 (4.84–8.75)	1.35 (0.85–2.13)	0.206	0.247
Form of TB					
Pulmonary	43	5.32 (3.94–7.17)	1.0	-	0.831
Extrapulmonary	22	6.42 (4.26–9.66)	1.21 (0.73–2.01)	0.456	
Pulmonary extrapulmonary and disseminated	8	5.14 (2.57–10.3)	0.99 (0.47–2.11)	0.987	
Previous treatment					
No	28	5.16 (3.56–7.47)	1.0	-	
Yes	44	7.25 (5.40–9.74)	1.38 (0.86–2.22)	0.178	0.286
Related to HIV^b^					
Opportunistic disease					
No	16	7.49 (4.59–12.2)	1.0	-	-
Yes	57	5.47 (4.22–7.09)	0.72 (0.42–1.27)	0.264	0.991
Use of ART					
Yes	57	5.16 (3.98–6.68)	1.0	-	-
No	18	8.48 (5.34–13.5)	1.53 (0.90–2.61)	0.113	0.315
CD4					
> 200 cells/mm^3^	43	3.78 (2.61–5.47)	1.0	-	-
< 200 cells/mm^3^	28	8.94 (6.63–12.1)	2.23 (1.39–3.60)	0.001	
No count	4	3.73 (1.40–9.94)	0.80 (0.28–2.30)	0.684	0.238

^a^ Proportionality test (Schoenfeld residual)

^b^Factors related to TB and HIV when initiating treatment for TB

**Table 6:** Association between death and defaulting from TB treatment in a cohort of PLHIV, adjusted analysis

Variables	HR adjusted (CI 95%)	*p*-value
Default from treatment for TB		
No	1.0	-
Yes	3.65 (2.28–5.83)	0.000
Alcohol consumption		
No	1.0	-
Yes. Light	0.13 (0.03–0.56)	0.006
Yes. Heavy/dependent	1.22 (0.62–2.43)	0.555
Smoker		
No	1.0	-
Yes	2.28 (1.33–3.89)	0.003
Form of TB		
Pulmonary	1.0	-
Extrapulmonary	1.56 (0.93–2.63)	0.091
Pulmonary, extrapulmonary and disseminated	0.99 (0.47–2.13)	0.992
CD4^a^		
> 200 cells/mm^3^	1.0	-
< 200 cells/mm^3^	2.39 (1.44–3.96)	0.001
No count	1.38 (0.45–4.23)	0.572

^a^Condition when initiating treatment for TB

## Discussion

The probability of death amongst people living with HIV who defaulted treatment for TB was almost four times greater than in those who did not. In addition, there was an independent association of death and CD4 count <200 cells/mm3, extrapulmonary TB and smoking. Light alcohol consumption was found to be a protective factor for death.

The study of risk factors for the default from TB treatment was well documented in a study by Maruza 2011, but there are few studies that have focused on the association between treatment default and death [18, 19, 21]. Moreover, we were unable to identify in the literature other studies that have addressed this topic in people living with HIV. In the present study we observed that the highest frequency of defaulting treatment occurred during the first quarter, which corroborates the work of several authors [16, 19, 21]. It is possible that patients present with some clinical improvement during this initial phase of treatment, and therefore the default rate is higher [16, 22]. However, early default is compatible with a lower chance of curing TB, with the risk of death being higher in the group of patients who defaulted treatment earlier (Q1) [21]. In the present study, deaths occurred at different moments in time (36% after 2 years), suggesting that the deaths may have occurred either due to the progression of TB or by the impact of a more prolonged duration of TB on the progression of HIV infection. Persistent infections, such as TB, may increase the patient’s viral load, which leads to a compromised immune function, lowering survival rates and increasing the chance of transmitting the HIV virus [10]. A high viral load may lead to a decrease in the CD4 count, through different mechanisms: accelerating cell loss induced by lysis of the virus or by destroying both healthy and infected cells, or even triggering apoptosis (cell death) of both healthy and infected cells [23]. In the present study it was observed that patients with a CD4 count of less than 200cells/mm3 presented a more than two-fold probability of death. This finding is in accordance with that described by other authors [13, 15, 24, 25] The CD4 cell count is a strong predictor for the progression of Human Immunodeficiency Virus - HIV [26] and patient survival. [27] Individuals with extrapulmonary TB presented a higher probability of death (borderline significance), which is in agreement with other studies. [28, 29] This association was explained by Kingkaew as being due to a delay in diagnosing this form of TB, for which there is a need to conduct more sophisticated tests in order to complete the diagnosis, which may delay initiation of treatment and thus jeopardize patient survival [29]. In the present study, it was observed that smoking presented a two-fold increase in the probability of death. This association has also been described by other authors [30, 31] and is attributed to a change in the structure and functioning of the lungs, as well as a decrease in immunity at both local and systemic levels [31]. Contrary to many studies, we found that light alcohol consumption was a protective factor for death, which is contrary to the findings of other studies that indicate an independent association between alcohol consumption and death [18, 32]. Perhaps light alcohol consumption encountered in our study as a protective factor for death signifies that light alcohol consumption is only a marker for improved conditions of health, and that this status in itself is a protective factor for death. The probability of death was higher in patients who were not using HAART but the difference did not reach statistical significance (*p* = 0.351), differing from a previou paper of our group [33]. It should be emphasized that most of the patients (81.1%) were using HAART. This study presented some limitations: the recruitment of patients to the study was uneven in the two reference services. Approximately two thirds of the study population was selected from Hospital Correia Picanço. This was due to logistical issues, as the fieldwork team was larger in this hospital. However, we do not believe that it may have nfluenced the association between treatment default and death. Another limitation was the exclusion of 192 patients for loss of recruitment, i.e., patients who started treatment for TB but were not included in the study. However, the exclusion of potentially eligible individuals was not due to any specific criterion and therefore it is unlikely that their absence distorted our results. In fact, for those patients (recruitment loss) it was possible to retrieve some information either from the SINAN or from the dataset of the Cohort AIDS PE and thus to compare patients enrolled and patients eligible but not enrolled. The two groups were similar in relation to age and sex but the default rate was higher among those enrolled in the study (data not shown). Although the default rate may have been overestimated we have no reason to think the association between default from TB treatment and death would differ in the two groups. As only 14.01% of the TB treatments started with a positive sputum smear, there may have been some misdiagnosis, i.e., some individuals without TB may have been classified as with TB However, it should be emphasized that professionals from both reference services have extensive experience in the diagnosis and treatment of tuberculosis, patients were followed until the end of tuberculosis treatment and patients for whom there was a change in the diagnosis of tuberculosis were excluded. The probability of death was greater for defaulters in the group with a positive, negative, or not performed sputum smear.

## Conclusion

The present study demonstrated an association between treatment default and death, with a higher probability of death when treatment was defaulted at an early stage. It is necessary to conduct further studies in order to assess the manner in which defaulting TB treatment affects the dynamics of the CD4 count over time, and the resulting effects of this dynamic on death. It would also be appropriate to compare the deaths that occur as a direct consequence of TB (TB death) with those that occur due to the impact of TB on the development of HIV/AIDS.

## Abbreviations

AIDS: Acquired immunodeficiency syndrome
ART: Antiretroviral therapy
BMI: Body mass index
CD4: Count linfocits T CD4
CI: Confidence interval
CNPq: Conselho Nacional de Desenvolvimento Científico e Tecnológico
DST: Sexually transmitted disease
HCP: Hospital Correia Picanço
HIV: Human Immunodeficiency virus
HR: Hazard ratio
HUOC: Hospital Univesitário Oswaldo Cruz
IC: Inform Consent
MDR-TB: Multidrug-resistant TB
MS: Ministry of Health
PE: Pernambuco
PLHIV: Peolpe living with HIV
PROCAPE: Pronto Socorro Cardiológico de PE
RECLINK: Probabilistic linkage program
SIM: Mortality information system
SISNEP: National system on research ethics
STATA: Data analysis and statistical software
TB: Tuberculosis
UFPE: Universidade Federal de Pernambuco
UNESCO: United Nations Educational, Scientific and Cultural Organization
UPE: Universidade de Pernambuco
